# Optimization of Cell Morphology Measurement via Single-Molecule Tracking PALM

**DOI:** 10.1371/journal.pone.0036751

**Published:** 2012-05-03

**Authors:** Nicholas A. Frost, Hsiangmin E. Lu, Thomas A. Blanpied

**Affiliations:** 1 Department of Physiology, University of Maryland School of Medicine, Baltimore, Maryland, United States of America; 2 Program in Neuroscience, University of Maryland School of Medicine, Baltimore, Maryland, United States of America; 3 Program in Molecular Medicine, University of Maryland School of Medicine, Baltimore, Maryland, United States of America; Stanford University School of Medicine, United States of America

## Abstract

In neurons, the shape of dendritic spines relates to synapse function, which is rapidly altered during experience-dependent neural plasticity. The small size of spines makes detailed measurement of their morphology in living cells best suited to super-resolution imaging techniques. The distribution of molecular positions mapped via live-cell Photoactivated Localization Microscopy (PALM) is a powerful approach, but molecular motion complicates this analysis and can degrade overall resolution of the morphological reconstruction. Nevertheless, the motion is of additional interest because tracking single molecules provides diffusion coefficients, bound fraction, and other key functional parameters. We used Monte Carlo simulations to examine features of single-molecule tracking of practical utility for the simultaneous determination of cell morphology. We find that the accuracy of determining both distance and angle of motion depend heavily on the precision with which molecules are localized. Strikingly, diffusion within a bounded region resulted in an inward bias of localizations away from the edges, inaccurately reflecting the region structure. This inward bias additionally resulted in a counterintuitive reduction of measured diffusion coefficient for fast-moving molecules; this effect was accentuated by the long camera exposures typically used in single-molecule tracking. Thus, accurate determination of cell morphology from rapidly moving molecules requires the use of short integration times within each image to minimize artifacts caused by motion during image acquisition. Sequential imaging of neuronal processes using excitation pulses of either 2 ms or 10 ms within imaging frames confirmed this: processes appeared erroneously thinner when imaged using the longer excitation pulse. Using this pulsed excitation approach, we show that PALM can be used to image spine and spine neck morphology in living neurons. These results clarify a number of issues involved in interpretation of single-molecule data in living cells and provide a method to minimize artifacts in single-molecule experiments.

## Introduction

Accurate measurement of cell morphology is critical in diverse realms of biology. In many cells, thin protrusions called filopodia transiently extend up to several microns from the cell as an essential step in cell motility, growth, or signaling [1]. In neurons, structures called dendritic spines house the components of signal transduction machinery at the synapse. The morphology of dendritic spines reflects function of the synapse within them [2], and their size and shape are regulated during changes in synapse efficacy [3]. Thus, their dimensions provide important and widely used indices of experience-dependent plasticity and diseases of the nervous system [4].

Observing such structures in intact cells requires optical techniques with resolution sufficient to discern details of interest. Dendritic spines are typically 0.5 to 2 µm in length, and their overall dimensions are commonly measured with confocal or multiphoton microscopy. However, the typical site of synaptic input is to the spine head, which is frequently <0.5 µm in diameter. In addition, the spine head is morphologically complex due to the presence of protrusive spinules and other specializations [3] that reflect a diverse set of functions taking place at spatially distributed positions within it [5], [6]. Importantly, the spine head is isolated from the main shaft of the neuronal dendrite by a thin neck similar in some respects to filopodia, up to 1 µm long but only ∼75 nm to 300 nm in diameter [3]. Spine necks are thought to influence synaptic transmission by a combination of electrical and chemical compartmentalization that is strongly dependent on their length and width [2], [7]. For these reasons, it would be of great interest to measure spine dimensions in live neurons. However, traditional far-field microscopy is not capable of resolving these structural details, and this problem is thus dependent upon super-resolution imaging techniques.

Single-molecule methods present a newly evolving opportunity to measure morphology of living cells simultaneously with the diffusive properties of molecules of interest *in situ*. Against a low-noise background, isolated fluorescent molecules can be localized with precision near 1 nm under ideal circumstances [8], [9], and precision better than 20 nm is now routine in cells [10], [11], [12]. This astounding capability has been exploited in a number of methods to visualize either molecular distribution or motion [13], [14]. A particularly powerful approach is termed single-molecule tracking photoactivated localization microscopy (PALM), in which GFP-type molecules photoconverted to a fluorescent state in very low numbers can be localized and tracked within the cell in a temporally iterative but spatially parallel fashion [15], [16]. Because it is essentially non-invasive, PALM provides an almost unique ability to measure single-molecule behavior of intracellular proteins without the use of fixation or antibodies [17]. However, the relatively small number of photons emitted by expressible photoconvertible probes compared to traditional dyes or quantum dots used in single particle tracking [18], [19] results in non-negligible localization precision or pointing accuracy, that is, the error in the calculated location of the probe [20]. Furthermore, the rapid bleaching of many of these fluorophores greatly reduces the number of image frames over which individual molecules can be tracked [15]. Thus, exploiting PALM to its fullest will require a careful understanding of how to extract the most information from the data.

In live cells, molecules are in motion during image acquisition. Many large cytosolic or membrane-associated proteins diffuse in the cell with an effective diffusion coefficient D_eff_ in the range of 0.1 to 1.0 µm^2^/s; unbound cytosolic proteins may diffuse much faster [21], [22]. In the ∼20 ms of a typical exposure during single-molecule tracking, such molecules undergo average displacements of ∼100 to 300 nm. Thus, molecular motion covers 10-fold greater scales than the localization precision, and presents a much larger problem that must be understood and minimized in order to measure molecule position and motion accurately.

For immobile molecules, localization precision is well understood to rely on the number of collected photons, the background noise, and the characteristics of the optical and detection system [20], [23]. However, this offers no direct insight to establishing how motion of the source contributes to degradation of precision. We therefore asked how motion of molecules affects the ability to localize them, how the magnitude of the localization precision affects our ability to measure molecular jump distances used to calculate D_eff_, and how the localization of moving molecules would affect our ability to render a high-resolution map of neuronal structures. Because preliminary experiments and published data indicated that many fluorescent proteins used for PALM will be bleached after imaging for only two frames, we paid particular attention to what could be deduced from two measurements of the same molecule's location.

## Results

To optimize live-cell, time-lapse morphology measurements, we reasoned that membrane probes are preferred over cytosolic probes for visualizing small structures such as dendritic spines, filopodia, or axons, because the surface-to-volume ratio of these structures is high, assuring a large number of probes to measure. Further, rapidly mobile probes resident in the plasma membrane would be most desirable, as their high diffusion coefficient would lead them to survey the cell surface quickly and enable the accumulation of a complete map of the cell surface in the least possible time.

**Figure 1:**
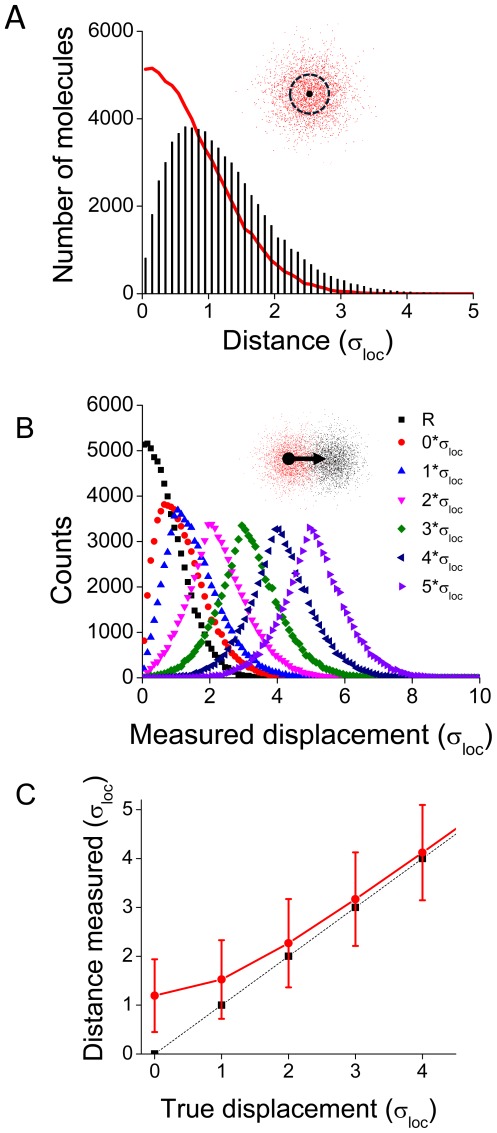
Masking of true motion by localization error. **A**. 10,000 single points representing localized positions of a molecule were generated randomly from a normal distribution with standard deviation σ_loc_. The distance of each point from the true location is plotted (**red line**). To simulate the effect of finite error on repeated localizations of a fixed object, a histogram of the distances between two randomly selected points within the same distribution is plotted as a histogram (**black bars**). **B**. Localized positions were randomly generated for pairs of molecules with true locations separated by increasing multiples of σ_loc_. The distance between random pairs of localized positions is plotted as a histogram for each true intermolecular spacing. **C**. The measured distance plotted as a function of true separation. Measured distances were larger than expected due to the non-trivial localization precision, but approached the expected measurement with increasing separation of the two true points.

Using expressed, fluorescently tagged proteins allows the rapid accumulation of many thousands of individual tracked molecules. However, currently available photoconvertible proteins photobleach rapidly, limiting the localization precision achievable in these experiments. We therefore first sought to determine the role of localization precision in determining the accuracy of our measurements of molecular motion. Our approach was to simulate possible localizations for a single molecule being imaged by an optical system with a resulting precision of 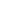
_loc_. Localizations were generated in a normal distribution centered at (0,0) distributed radially from 0 to 2π. The distribution of 100,000 individual localized positions from the origin is plotted as a histogram (Fig. 1A). The average distance from the true location of the molecule was 0.80±0.6 *
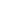
_loc_. To simulate the apparent motion that would be measured when tracking a fixed molecule in two consecutive frames, a second point was randomly generated in the same fashion; the distance between two points within the distributed localizations for a fixed molecule is displayed as a histogram (Fig. 1A) with a broad peak from 0.65–0.85 *
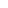
_loc_ and was on average 1.2±0.77 *
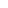
_loc_.

As localization error alone contributes a minimum apparent motion of ∼1.2*
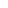
_loc_, we sought to determine the role of localization precision in the ability to measure molecular motion. To do so, we selected pairs of possible localizations of two molecules separated by various distances (in terms of 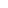
_loc_). The distribution of distances between pairs demonstrates a high degree of overlap between unshifted molecules and molecules separated by 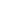
_loc_ (1.2±0.77 vs 1.53±0.71*
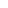
_loc_), and decreasing overlap with increasing separation (Fig. 1B). The localization precision introduced a systematic error in measured distance so that on average, measured distance between localizations was greater than the actual distance between pairs of molecules. The high degree of error in measured distance between subsequent localizations of the fixed molecule decreased markedly as the distance of separation increased, as evidenced by the convergence of the mean distance between localized points plotted against the true displacement of the localized molecules (Fig. 1C).

**Figure 2:**
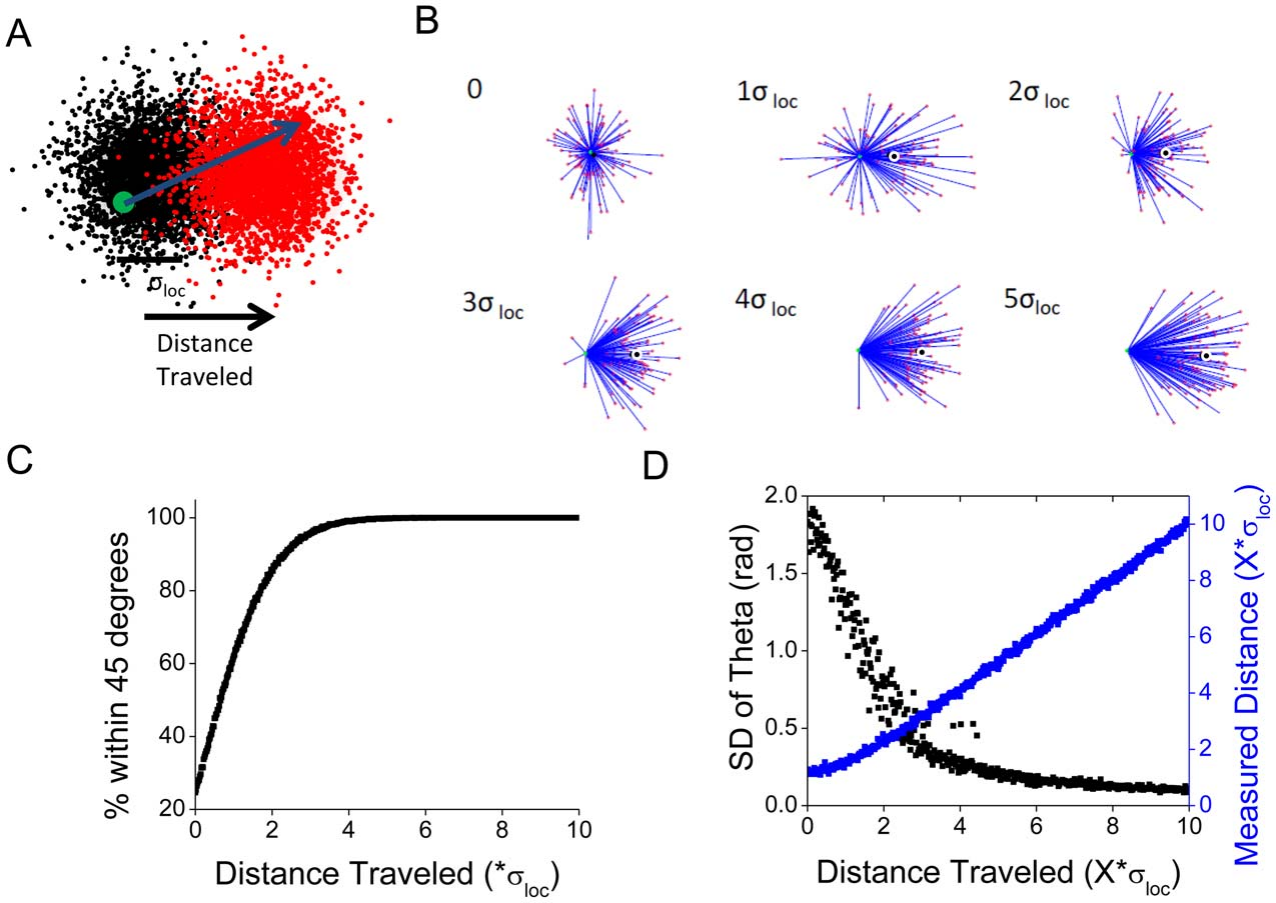
Accurate determination of direction depends on localization precision. **A**. Monte Carlo simulations were used to determine the distance of motion required to accurately determine the direction of a moving molecule localized with precision of σ_loc_. 100 pairs of single points were generated in normal distributions with standard deviation σ_loc_ centered around two points separated by increasing distance. **B**. Pairs were plotted as a compass plot with the initial point (green) in the center connected to the second point (red) by a blue line. The net distance traveled parallel to the real translation of the distribution is marked by the black dot. **C**. The percent of vectors pointing toward the correct quadrant (within 45 degrees of the correct direction). **D**. The measured distance between localized points (blue) and the standard deviation of θ for the accompanying vectors (black). Each dot represents mean of 100 paired measurements.

We utilized a similar approach to determine the effect of localization precision on our ability to confidently determine directionality of motion. As before, sets of localized positions were generated surrounding simulated molecules in a normal distribution with a standard deviation of 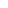
_loc_. Respective points were separated by 0 to 10* 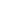
_loc_. Figure 2B shows examples of 100 points drawn so that a vector (blue line in Fig. 2A) connects the first localized position (center, green dot) to the second localized point (red dot). As expected, for non-moving points, the angle θ of motion from the first localized position to the second position was distributed equally from 0 to 2π. With increasing separation between the actual position of the two points, the vectors became increasingly oriented to the real direction of motion, so that at a separation of 1*
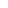
_loc_ 62% percent of vectors were within π/4 accuracy, and at a separation of 2*
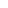
_loc_ 85% of the vectors were within the correct quadrant. This is quantified in Figure 2C, which demonstrates the rapid increase in the number likelihood that θ is within 45 degrees of the direction of true motion, and in Figure 2D, which demonstrates the rapid decrease in the variance of θ (black dots, average of 100 pairs) as the mean distance measured increases (blue dots, mean of 100 pairs).

**Figure 3:**
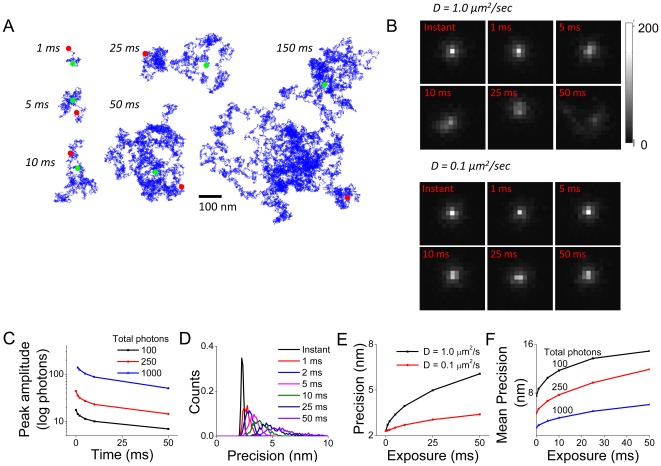
Effect of motion during image acquisition on single molecule photon distribution and localization precision. **A**. Examples of random walks taken by a molecule over various timescales. Blue lines depict the path taken by the molecules, with green and red dots denoting the starting and ending position, respectively. **B**. Examples of photon distributions emitted from moving molecules of the indicated D over integration times ranging from 0 to 50 ms. **C**. The mean value of the brightest pixel (N = 1000 molecules) is plotted against integration time for molecules emitting 100, 250, or 1000 photons over the course of the integration time. **D**. Histogram of calculated precisions for molecules with D = 1.0 µm^2^/sec. **E**. The mean calculated precision for molecules with D = 0.1 µm^2^/sec (**red line**) and D = 1.0 µm^2^/sec (**black line**). Points represent mean of 1000 molecules. **F**. To examine the interaction of movement-induced error with photon-dependent precision, the mean error of molecules emitting 100 photons (**black line**), 250 photons (**red line**), and 1000 photons (**blue line**) were plotted as a function of exposure duration.

We directly evaluated the effect that motion would have on the calculated localization precision of single molecules. To do this we performed Monte Carlo simulations of images captured of single molecules allowed to travel by random walk in two dimensions over various timescales. Molecules took a calculated number of randomly oriented steps (Fig. 3A), with assorted complicated resulting trajectories. To simulate the resulting image that single molecules would form on the image plane during acquisition, 1000 photons were emitted in a normal distribution (σ = 250 nm) at evenly distributed times during the exposure. Representative single frames clearly demonstrate that degradation of the image occurs with as little as 5 ms exposure for molecules with diffusion coefficients of 1.0 or 0.1 µm^2^/s, with substantial distortion of the PSF occurring for the faster molecule and at longer exposures (Fig. 3B). This was most clearly measured as a decline in the amplitude of the brightest pixel of the image (Fig. 3C).

To determine the effect of this distortion we calculated the localization precision for 1000 generated molecules (D = 1.0 µm^2^/s) moving over timescales of 0 to 50 ms. We set the detection threshold to a very low level to allow detection of peaks at the longer exposure times; these molecules would be undetectable in the presence of noise. In addition, in many cases quickly moving molecules diffusing over long exposures were detected as more than one peak; in these cases only the brightest peak was fit and localized. The distribution of calculated precision was broader and shifted to greater values with increasing exposure times (Fig. 3D). Precision was degraded for both quickly moving and slower moving molecules as exposure time increased (Fig. 3E) and for molecules of varying photon output (Fig. 3F).

**Figure 4:**
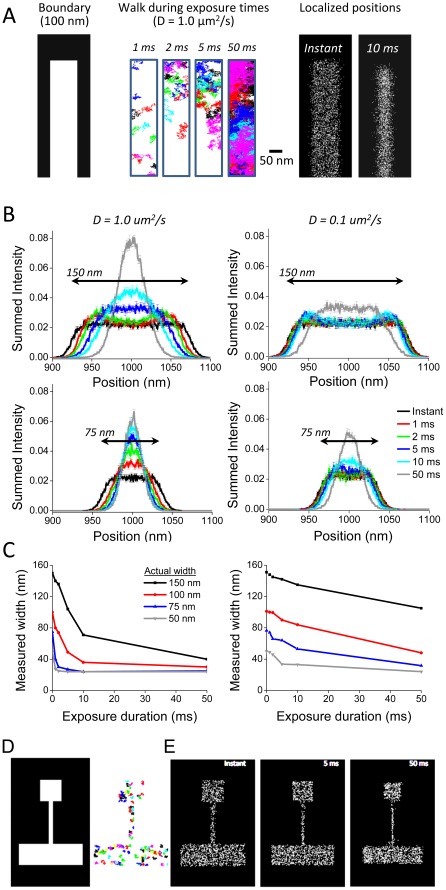
Measurement of morphology is degraded by molecular motion during prolonged integration times. To determine the effect of diffusion on the measurement of cell structure based on the position of localized molecules, we simulated molecules diffusing within a bounded space analogous to a filopodium. **A**. Random walks of molecules with D = 1.0 µm^2^/s were generated within a rectangle 100 nm wide (**left, center**). The localized position of the molecules is displayed for simulated acquisition using integration times of 0 (i.e., a fixed particle) and 10 ms (**right**). **B**. Molecules with D = 1 µm^2^/s (**left**) or 0.1 µm^2^/s (**right**) began their walks at random initial points within the bounded rectangle as in A. The density of localized positions across rectangles 150 nm in width (**Top**) or 75 nm (**Bottom**) plotted as histograms for exposures ranging from 0 to 50 ms are shown. **C**. The half-width of the bounded regions is quantified for D = 1.0 µm^2^/s (**Left**) or 0.1 µm^2^/s (**Right**). **D**. The effect of motion on the distribution of localized positions within a spine was modeled using a region consisting of a 500 nm square spine head and a neck that was 1000 nm long and 100 nm wide connected to a dendrite that was 500 nm wide and 1500 nm long (**left**). Plots of the paths taken by individual molecules with D = 1 um^2^/s and an exposure duration of 1 ms are shown (**second panel**). **E**. Localized positions of simulated imaged acquired using integration times of 0 (fixed particle), 5, and 50 ms are shown. Note the degradation of morphological accuracy with long exposure times.

We sought to determine the effect of motion artifacts on other parameters, including our ability to define structures and diffusion kinetics of moving molecules. We first used Monte Carlo simulations to determine the effect of diffusion during increasingly long exposures on the appearance of bounded structures. Molecules were placed randomly within a rectangular space and underwent random walks representing diffusion over exposures ranging from 0 to 50 ms (Fig. 4A, left). Over short integration times, each molecule's motion was confined to small regions of the bounded space; with increasing integration times molecules were able to span very large portions of the region. Each molecule emitted 1000 photons and was localized. The distribution of localized positions demonstrated a clearly thinner appearance to the rectangle generated from 10 ms exposures than that representing fixed molecules (Fig. 4A, right). This difference was quantified as measuring the full width at half maximum of histograms of the molecular density. Longer integration times resulted in increasingly sharply peaked distributions (Fig. 4B), with decreasing half-widths (Fig. 4C). We applied this same logic to simulated spine-shaped objects. Spines were generated as bounded regions consisting of a 500 nm square head, a 100 nm wide neck, and a dendrite (Fig. 4D). As above, longer integration times resulted in preferential localization of molecules away from the boundaries of the region (Fig. 4E); this resulted in a clear decrease in apparent width of the spine neck as well as the area of the spine head.

**Figure 5:**
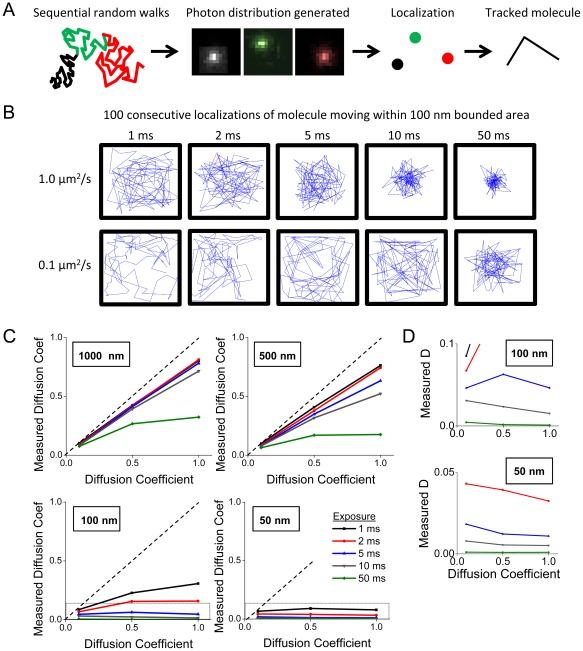
Short exposure time is critical for accurate measurement of diffusion for molecules within bounded space. To determine the effect of exposure duration on measurement of diffusion of a freely moving molecule, we simulated molecules moving within bounded spaces analogous to small cellular compartments of interest. **A**. Molecular trajectories were generated as a series of 100 concatenated random walks. During each walk, each molecule generated an average of 1000 photons, creating an image that could be fit to localize the molecule for a single time point. Tracks were generated from sequential positions of the molecule. **B**. Tracks plotted for 100 sequential localizations of a molecule with diffusion coefficient of 1.0 µm^2^/s (**top**) or 0.1 µm^2^/s (**bottom**) moving within a bounded region represented by a 100 nm box. The measured path of tracked molecules was clearly affected at longer exposures, as localized positions progressively approached the center of the square. The resulting measured D_eff_ was subsequently decreased from the expected value. **C**. Mean D_eff_ for molecules of various D are plotted for several exposure times in regions of decreasing size. **D**. The Y axis of the final panels from C is expanded to demonstrate that within the bounded space, faster molecular motion results in decreased measured diffusion. This effect is reduced with rapid exposure times.

A substantial advantage of single molecule tracking of photoconvertible fluorophores is that the motion of the molecule can be measured with simultaneous rendering of super-resolved images. We sought to examine the effect of integration time on the (measured) D_eff_ of single molecules diffusing within restricted regions. Single molecule tracks were generated as a concatenated series of random walks within bounded regions (Fig. 5A). With increasing integration time, localized positions were increasingly biased toward the center of the bounded space (Fig. 5B). Diffusion coefficient was calculated based on the mean distance between localized positions (D = R^2^/4t), where R is the distance and t is the time between localized positions, and plotted as the mean of 7 molecules tracked over 1000 frames each (Fig. 5C, 5D). Measured diffusion coefficients decreased for all molecules as the size of the bounded region decreased; this effect was especially pronounced for faster molecules integrated over longer exposure times. This effect was particularly marked in 50 and 100 nm bounded regions, in which the measured diffusion coefficient actually decreased with faster moving molecules (Fig. 5D). Thus, careful attention must be paid to exposure time when measuring diffusion in bounded regions.

The previous considerations suggest that an optimal acquisition paradigm will depend on the diffusion characteristics of the probe of interest as well as the time scale required for morphology analysis. To achieve the most accurate morphology in a short time, we developed a simple approach that is flexible enough to adapt to diverse needs. To design the acquisition protocol for live-cell experiments, we considered the duration of excitation (t_e_). In typical single-molecule tracking experiments, excitation persists for the duration of the image acquisition, and the acquisition speed is limited by the readout speed of the camera. For standard EM-CCDs such as used here, the frame rate at a full 512×512 pixel frame is 30 Hz t_e_ = 33 msec; a reduced acquisition window is frequently used to increase frame rate to 50 Hz, t_e_ = 20 msec. Further reduction of t_e_ requires unacceptable limitation of the readout area of the EM-CCD. Thus, we sought to illuminate molecules for only a brief proportion of each frame, while maintaining a larger imaged region of the CCD and a frame rate that could be set independently. To do this, we synchronized the initiation of the camera frame (provided through the “fire” TTL pulse on Andor's EM-CCDs) to a TTL timing source (in this case, an AMPI Master-8) which gated an AOTF controlling the excitation laser. In this manner, both the exposure time and the delay after the start of the frame could be varied freely. Short illumination times required high-intensity excitation, which we achieved by expanding the incident collimated laser beam only to ∼2.2 µm before focusing it onto the back focal plane of the objective for oblique (near-TIR) illumination. With this scheme, we could achieve either high acquisition frame rates (routinely 100 Hz of 15×50 µm at 100 nm per pixel), or short t_e_ (routinely 0.5 to 10 ms), or both.

**Figure 6:**
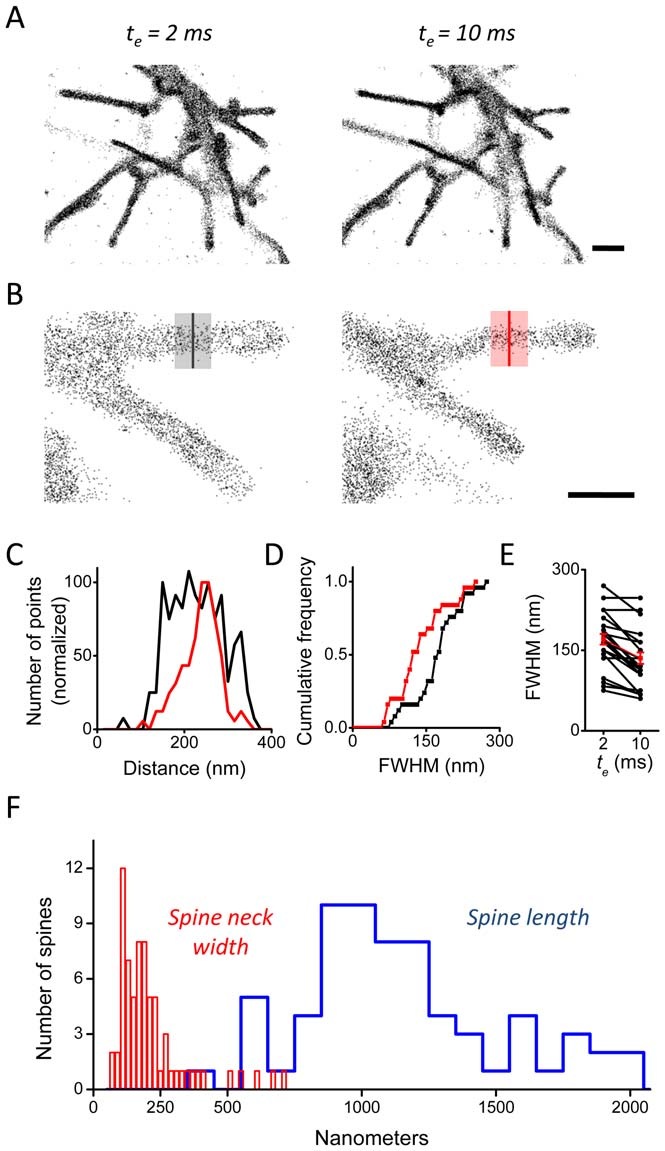
More accurate morphology of living neurons using short, pulsed excitation during acquisition. **A**. Cultured hippocampal neurons grown 10 days in vitro (DIV) expressing membrane-mEos2 were imaged at 50 Hz using excitation pulses of two durations (t_e_ = 2 ms and 10 ms) delivered in random order. The distribution of localized positions was plotted (A, enlarged in B), demonstrating a thinner appearance of neuronal processes imaged with longer t_e_. **C**. Intensity profile of line scans drawn perpendicular to the neuronal process as in B. **D**. Cumulative frequency plot of the line scan full width at half maximum intensity. **E**. Paired comparison showed that the width of the processes was consistently diminished in the longer exposure. **F**. Measured spine neck widths (red) and spine lengths (blue) in neurons grown 11 to 12 DIV. Neurons were imaged at 50 Hz for 10,000 frames with t_e_ = 4 ms.

Using this configuration, we acquired sptPALM images of filopodia, spines, and spine necks of cultured neurons expressing membrane-mEos2. This molecule is targeted to the plasma membrane by virtue of a palmitoylation motif, and is quickly mobile (D_eff_>1 µm2/s; data not shown). To test whether shorter exposure times permitted more accurate reconstruction of cell morphology, we set t_e_ at 2 or 10 ms (in randomized order) while imaging the same field of view at a fixed 50 Hz image acquisition rate. To compare as directly as possible to our simulations, which maintained a constant photon output per frame, we altered the laser power L in concert with t_e_ to maintain equivalence of L* t_e_. We plotted locations of all molecules meeting criteria (see Methods), which was sufficient to provide clear delineation of apparent cell morphology at each t_e_. Visually, the influence of t_e_ on the diameter of fine processes was clear (Fig. 6A, 6B). To quantify the effect, we measured filopodia, whose nearly constant diameter along their length makes their apparent diameter straightforward to ascertain. We measured the full width at half-maximum of N processes at the two t_e_ values (Fig. 6C–E). Indeed, the shift toward larger widths with reduced t_e_ is clear evidence that it more accurately captures cell morphology at scales relevant to super-resolution imaging. To demonstrate the utility of the approach with a structure of subdiffraction dimensions, we next imaged dendritic spines in cultured hippocampal neurons. Utilizing a small t_e_ = 4 ms, we measured a large number of spines with a median neck width of 179 nm (N = 71 spine necks) (Fig. 6F).Spine neck diameter as measured by electron microscopy is quite variable but generally less than 200 nm, for instance with reports of 150±60 nm [24], 40 to several hundred nm [25], and 200±40 nm [26]. Stimulated emission depletion microscopy in hippocampal slices revealed spine neck diameters of ∼75 nm to 230 nm [27]. Published data using a single-molecule tracking approach but utilizing a large t_e_ of 20 ms [28] measured a neck diameter of 90±40 nm, consistent with the smaller value expected based on our modeling.

## Discussion

In living cells, the high precision of single-molecule localization in PALM permits both the rendering of highly resolved maps of molecular distributions [11], [16], [29], [30] and massively parallel motion analysis of individual molecules [15], [17], [31], [32]. In this paper, we explored factors limiting ability to accurately define the motion of single molecules. Most fundamentally, localization precision limits the accuracy of the distance and direction measured for tracked particles. Furthermore, the motion of the molecules during single exposures of image acquisition results in blurring that reduces the definition of imaged peaks—reducing the localization precision. Beyond direct effects on localization precision, we demonstrate that molecular motion within a bounded region results in a distribution of localized peaks that is increasingly inwardly biased as integration time is increased. Thus, either short image integration times or the imaging of slowly moving molecules are necessary to accurately define the edges of bounded regions. We propose one straightforward solution to this problem, and demonstrate that by utilizing short excitation pulses synchronized to the camera exposure, rapidly diffusing membrane-bound mEos2 can be imaged to measure the morphology of filopodia, spines, and spine necks of live neurons.

Our work highlights a number of factors critical to experimental design in single molecule tracking PALM. The two-dimensional distribution of positions of a molecule localized with precision σ_loc_ results in a distribution of errors such that on average, molecules are localized ∼0.8*σ_loc_ from the true location of the molecule. This error is propagated in each measurement so that repeated measurements of the same, unmoving molecule are on average ∼1.2*σ_loc_ apart. In single-particle tracking, this results in a significantly increased error in calculation of distance traveled for molecules moving less than σ_loc_ between acquisitions, which defines a practical threshold for detectable motion. Only as the distance traveled increases beyond ∼2*σ_loc_ does the distribution of measured distances become reliably different from that of a fixed molecule. Similarly, the accuracy of the angular component of vectors drawn between sequential localizations of a moving molecule dramatically increases at ∼σ_loc_. This has little impact on experiments utilizing very bright and stable fluorophores such as quantum dots that can be localized many times with high precision. However, this is likely to become important in many experiments using currently available photoconvertible proteins such as mEos2 [18] or Dendra [33] where localization precision is limited by the low brightness of the molecules and where photobleaching limits the duration of trajectories. Aside from optimizing imaging and analysis to achieve precise localization, this limitation can be overcome by using either using rapidly moving molecules or by inserting sufficient inter-frame pauses to allow molecules time to diffuse >2*σ_loc_ [17]. Purely measuring large numbers of molecules cannot overcome these limits.

Although diffraction limits the ability of light microscopes to resolve features less than approximately 200 nm apart [34], [35], single-molecule localization permits the determination of molecular position with much finer precision. Various methods have been developed to estimate the localization precision in single molecule experiments [10], [20] and have been useful in guiding experimental design. These works have stressed the number of acquired photons in the object, the background noise, pixilation artifact, and the quality of the optical system. Studies on diffusion of single molecules within corrals have revealed the importance of sampling rate as well as the importance of proper sampling density [36]. Indeed, we predict, as others have shown [37], that increased acquisition rate generally increases the accuracy of D_eff_ measurements. However, aside from increasing the number of localizations from which the map of the cell is reconstructed, increasing acquisition speed independent of t_e_ is not expected to better resolve cell morphology. The resolution that can be confidently achieved for structures within images rendered from localized positions has indeed been shown to be directly related to the density of localized molecules [31].

Single-molecule blurring can be severe during acquisition, even using exposure times of 30 ms. Indeed, under the right conditions, this property can be used to exclude freely diffusing molecules entirely because their peak amplitude is reduced below detection threshold [17], effectively isolating bound or nonmoving populations of molecules. Because localization precision establishes the limits of accurately defining molecular motion, we sought to determine the effect that molecular motion exerts on our ability to localize moving molecules. We examined the effect of motion with a range of diffusion coefficients (0.1 to 1.0 µm^2^/s) relevant to single particle tracking experiments, during commonly used camera exposure durations (1 to 50 ms). This range of diffusion coefficients covers a wide range of biological molecules commonly targeted in particle tracking experiments, from ion channels and other transmembrane molecules to membrane-bound signaling molecules. Not surprisingly, blurring of rapidly moving molecules resulted in both a reduction in the amplitude of imaged peaks, and a corresponding reduction in localization precision. We found in addition that within bounded regions, the motion of molecules away from diffusion barriers during the acquisition of a single image adds a further confounding factor, in that the distribution of localized positions is shifted increasingly inward as the speed of motion increases or the exposure duration lengthens. This complicates the measurement of the size of such bounded regions and of molecular motion within them, and can result in observations likely to be misinterpreted. For instance, within small regions molecules moving quickly can exhibit slower *measured* diffusion than their truly slowly-moving counterparts, because each measurement essentially represents the average of a long trajectory that covers much of the region.

These errors can be easily avoided if imaging only slowly diffusing molecules. However, this benefit must be weighed against the disadvantage of requiring increased acquisition times to provide sufficient time for thorough determination of either molecular behavior or cell morphology. We desired a method that combined both rapid acquisition and accurate measurement of molecular motion, and demonstrate that decreasing the integration time during which images of quickly moving molecules are acquired both increases the localization precision and reduces artifacts at the edges of bounded regions. To accomplish this we utilized a system in which gating of the excitation source was synchronized to the initiation of the camera exposure, but could be varied independently to allow sub-millisecond excitation combined with imaging of wide spatial regions. The measured width of cell protrusions varied inversely with the integration time, consistent with the predictions of our modeling. Neuronal processes artifactually appeared more slender than expected based on electron microscopy even with exposure times of 10 ms, which are themselves shorter than 50 Hz acquisitions frequently used in single molecule imaging [15], [28], [38], [39], [40]. While in principle the briefest possible light pulses will reduce motion artifacts to the greatest extent, a practical limit is reached when considering the power available from affordable lasers. We found that 2 msec excitation pulses offered an excellent compromise for measuring diffusion of membrane-mEos2 while mapping neuronal morphology. Assuming 5000 frames are required to gather a sufficiently high density of molecules to resolve morphology, such images could thus be acquired once per 10 sec in time lapse, providing excellent utility for live-cell super-resolution microscopy.

Our simulations were conducted as 2D random walks, and our experiments calculated the 2D best-fit location. When single-molecule tracking of intracellular proteins in 3D becomes more routine (though it is possible now with quantum dots and a limited selection of other probes, [41], [42]), it will be useful to extend our results to three dimensions. Such extension will presumably facilitate more accurate measurement of morphology. Indeed, recent work has demonstrated that the error of calculating D_eff_ from 3D trajectories projected to 2D can be as high as 25 to 50%, and that tracking in 3D can decrease this error if the imaging frame rate is high compared to the molecule mobility [39]. Our findings suggest that this benefit would be much greater and easier to achieve if the excitation duration as well as the imaging frequency is taken into account. Notably, though we concentrate here on deriving cell morphology from the localization of large number of photoconvertible GFPs using PALM, a complementary approach of using a small number of quantum dots each tracked for numerous frames can also be used [28], [39]. In each case, reducing the exposure duration will improve accuracy of the measured localization, D_eff_, and cell morphology.

## Materials and Methods

### Ethics statement

Animals were used in full compliance with the National Institutes of Health/Institutional Animal Care and Use Committee guidelines. The protocol was approved by the Institutional Animcal Care and Use Committee of the University of Maryland School of Medicine Office of Animal Welfare Assurance, under protocol #0111001.

### Simulations

Monte Carlo simulations were performed in Matlab (Mathworks; Natick, Ma). To simulate the repeated localization of a single molecule with localization precision σ_loc_, two-dimensional distributions of positions were generated with their distance from the origin normally distributed with standard deviation equal to σ_loc_, and their radial position evenly distributed from 0 to 2π. Random walks were generated so that molecules took an appropriate number of steps, N_steps_, in random directions so that N_steps_ = 4Dt/r^2^, where D is the diffusion coefficient of the molecule, t is the integration time, and r is the mean step size. At each step, the displacement in x and y was a randomly generated number ranging from −√2 to √2*r, so that the mean step size approached r when N_steps_ was large. For all simulations, r was set to 1 nm. For molecules moving within bounded regions, steps that would have resulted in exit from the region were recalculated until the position was within the boundaries.

To distribute emitted photons equally across the positions occupied by a moving molecule during integration of a single camera exposure, the probability that a molecule generated a photon at a given step was given by the number of photons divided by the number of steps taken by the molecule during the integration time. The position of each emitted photon was chosen from a set of random, two-dimensional positions generated as a normal distribution of random points with standard deviation equal to the simulated point spread function (PSF) of the microscope *s*, evenly distributed radially from 0 to 2π, and centered at the molecule's true position at each step. Final photon distributions were then accumulated in a grid of 100 nm pixels and localized using the best fit of a two-dimensional elliptical Gaussian as previously described [17]. To simplify analysis and prevent localization failures, we generally omitted background noise. Localization precision was calculated as described [20] so that *σ* ^2^ = (*s* ^2^+*a* ^2^/12)/*N*+8π*s* ^4^ *b* ^2^/*a* ^2^ *N* ^2^, where *a* represents the pixel size of the imaging detector, *N* represents the number of photons in the molecule, and *b* represents the background noise. Note that in the absence of noise, the second term is dropped.

### Hippocampal cell culture and transfection

Dissociated hippocampal neuron cultures were prepared from E18 rats as previously described [43]. Glass coverslips (Warner Instruments) were washed for 3 hours in ammonium hydroxide∶hydrogen peroxide: water mixture at a ratio of 1∶1∶5 and flamed with methanol. 50 nm gold beads (Microspheres-Nanospheres, 790116-010) were applied as previously described [17] and coverslips were coated with Poly-D-Lysine overnight prior to plating. Transfections were performed using Lipofectamine 2000 on the specified days and imaging was performed 24–48 hours later. mEos2 was a gift from Sean McKinney. Membrane-mEos2 was constructed based on the sequence of EYFP-Mem (Clontech), by appending to mEos2 the N-terminal 20 amino acids of GAP43, which contain a palmitoylation motif.

### Imaging

Imaging was conducted on an Olympus IX81 inverted microscope with a 100X/1.45 Plan Apo oil immersion objective. Illumination was provided by 561 nm (150 mW) and 405 nm (100 mW) diode lasers which were expanded to ∼2.2 µm and entered the epi-illumination port of the microscope where they were focused on the back focal plane of the objective for oblique (near-TIR) illumination. AOTF gating of excitation lasers was controlled by a TTL timing source (AMPI Master-8) which was synchronized to the initiation of the camera frame by the “fire” TTL pulse on the Andor iXon DV897ECS-BV backthinned EM-CCD. In this manner, excitation pulse length (integration time) could be independently varied from the exposure time. With this scheme we could achieve both high acquisition rates (routinely 100 Hz of 15×50 µm regions at 100 nm per pixel) coupled to variable excitation times as short as 0.5 ms. Imaging was performed at room temperature.

10,000 frames were captured at 50 Hz or 100 Hz using 2, 4, or 10 ms pulses as specified. Laser intensity was calibrated so that integrated excitation per frame was the same. Single molecules were fit to a two-dimensional Gaussian and localized in MATLAB as described previously [17]. Plots of the distribution of localized molecules were further processed using ImageJ. Linescans were drawn through spine necks and the base of protrusions and the halfwidth of the density was measured in Origin.
